# Red Blood Cells Mean Corpuscular Volume (MCV) and Red Blood Distribution Width (RDW) Parameters as Potential Indicators of Regenerative Potential in Older Patients and Predictors of Acute Mortality – Preliminary Report

**DOI:** 10.1007/s12015-020-09977-6

**Published:** 2020-05-05

**Authors:** Katarzyna Brzeźniakiewicz-Janus, Joanna Rupa-Matysek, Andrzej Tukiendorf, Tomasz Janus, Mirosław Franków, Marcus Daniel Lancé, Lidia Gil

**Affiliations:** 1 Department of Hematology, Multi-Specialist Hospital Gorzów Wielkopolski, Faculty of Medicine and Health Science, University of Zielona Góra, Gorzów Wielkopolski, Poland; 2 Department of Hematology and Bone Marrow Transplantation, Poznań University of Medical Sciences, Poznań, Poland; 3 Department of Public Health, Wrocław Medical University, Wrocław, Poland; 4 Department of Forensic and Clinical Toxicology, Pomeranian Medical University in Szczecin, Szczecin, Poland; 5 Department of Anesthesiology, Intensive Care Unit and Perioperative Medicine, Hamad Medical Corporation, Doha, Qatar

**Keywords:** MCV, Mean corpuscular volume, RDV, Red blood distribution width, Acute mortality, IGF-I, GH, VSELs

## Abstract

This study presents the statistical results of patients who had been recently discharged from hospital within one month after their treatment in the emergency department (ED). Using routine (14,881) MCV and RDW measurements and statistical tools, we could predict acute mortality in these patients (*N* = 1158), adjusted for age. It is likely that an increase in the MCV and RDW parameters may correlate in some of our older patients with a poor prognosis with an increased level of circulating IGF–I, which affects red blood cell parameters. The research presents the prognostic statistics of the analyzed clinical factors as well as speculates on the potential correlation of these parameters with the regenerative potential of stem-cell compartment. Analysis shows that both MCV and RDW are statistically significant (Area Under Curve [AUC], lower CI 95% >50%) predictors of acute mortality in ED patients. The classification of patients based on their MCV threshold (= 92.2 units) indicates a proper clinical prognosis in nearly 6 of 10 subjects (AUC >58%), whereas taking into account RDW (=13.8%) indicates a proper clinical prognosis in no more than 7 of 10 individuals. The report concludes that by employing strongly fitting (95%) quadratic modeling of the ORs against the biomarkers studied, one can notice a similar relationship between MCV and RDW as diagnostic tools to predict regenerative potential and clinical outcomes in older patients. Although RDW alone had a 10% higher diagnostic value in terms of predicting early death in the emergency department in patients that were admitted to the ED and subsequently hospitalized, also taking the MCV measurement improved accuracy in predicting clinical outcomes by 2.5% compared to RDW alone.

## Introduction

Recently, mean corpuscular volume (MCV) as a measure of the average size of erythrocytes has been associated with mortality in several clinical settings. Elevated MCV (generally >100 fL) is often characteristic of underlying conditions, such as nutritional deficiencies, drug and alcohol use [1], vitamin B12 deficiency [2], certain medications, or bone-marrow disorders [3]. In patients with stage ≥3 chronic kidney disease (CKD), MCV was associated with all-cause mortality, cardiovascular disease mortality, and infection-associated mortality [4]. Performing a Cox regression analysis, [5] showed MCV to be a predictor of composite cardiovascular (CV) events in CKD patients as a major confounding factor. Based on the >100,000 incident haemodialysis (HD) patients and those with higher (>98 fL) MCV levels, a higher all-cause, cardiovascular, and infectious mortality risk was also confirmed by Dratch et al. [6]. A gradient relationship between increased MCV and deaths associated with cerebral ischemic stroke (CIS) and ischemic heart disease (IHD) was reported by Taiwanese investigators using a large-scale (66,294) population-based study [7].

MCV is related to another parameter: red blood distribution width (RDW), a component of the complete blood count indicating the size heterogeneity of the erythrocytes circulating in peripheral blood. Mathematically, the RDW is calculated using the following formula: RDW = (standard deviation of MCV ÷ MCV) × 100. In elegant studies, a higher RDW in patients has been associated with a shorter life span [8, 9]. Based on this, it has been postulated that RDW may correlate with a higher level of circulating insulin, like growth factor-1 (IGF–I), and an increased level of IGF–I negatively correlates with patient life span [10, 11]. IGF–I is a known stimulator of erythropoiesis and erythrocyte maturation, and thus RDW indirectly reflects an elevated IGF–I level in peripheral blood, which explains a link among IGF, MCV, and RDW [12]. Therefore, it has been proposed that patients with higher circulating IGF–I levels and a shorter life span have higher RDW [8, 9].

In this short report, we present the statistical results of patients who had been recently discharged from the hospital within one month after their treatment in the emergency department (ED). Using routine MCV and RDW measurements and statistical tools, we could predict acute mortality in these patients adjusted for their age. We present prognostic statistics of the analyzed clinical factors as well as speculate on the potential correlation of these parameters with the regenerative potential of stem cell compartment.

## Materials and Methods

We conducted a single-center retrospective study from the MCV clinical records in the emergency department (ED) at the Multi-Specialist Hospital in Gorzów Wielkopolski, Poland. Patients were included if at study entry they: (1) received care between January 1, 2016 and December 31, 2019, (2) were 18 years old or older, and (3) had at least one biomarker measurement (for entry we examined the data from a total of 23,992 patients with 34,929 laboratory tests). In the study we focused on the chronic nature of the diseases and discarded the urgent characteristic of clinical events. According to the International Statistical Classification of Diseases and Related Health Problems, 10th Revision (ICD–10), we analyzed patients from the A to N classes, excluding from the study those with O, P, Q, R, S, and T diagnoses. Since repeated visits of patients to the ED and their subsequent treatment might affect the MCV indices, we examined only the first laboratory tests of a biomarker during the studied period. In total, the analysis comprised 14,881 patients whose first MCV/RDW measurements were recorded in the Emergency Department within the period 2016–2019, with a subsequent 1158 one-month, all-cause mortality observation (MCV and RDW measurements were performed in the hospital’s Laboratory Unit using Sysmex XN-2000, Sysmex Corporation, Japan, analytical systems using EDTA-KE/2.7 ml samples).

The main descriptive statistics of ED patients, according to the criteria described for sex and ICD–10 classes of the first diagnoses, are reported in Table 1.

**Table 1:** Main descriptive statistics of ED patients according to sex and ICD10 classes of the first diagnoses

Sex:	Males			Females		
ICD–10 class	# of diagnoses	# of deaths(%)	Age ± st. dev.	# of diagnoses	# of deaths(%)	Age ± st. dev.
gastroenterological	804	37(5%)	55.9 ± 17.4	624	39(6%)	61.5 ± 20.2
infections (virus, bacterial, fungal)	33	0(0%)	52.1 ± 17.0	31	2(6%)	60.7 ± 22.6
cardiological	3513	359(10%)	65.2 ± 13.4	3049	343(11%)	72.8 ± 13.6
metabolic	283	12(4%)	62.2 ± 18.0	311	32(10%)	71.9 ± 17.4
urogenital	484	18(4%)	56.0 ± 19.0	421	28(7%)	59.9 ± 21.4
neurological	565	5(1%)	52.7 ± 17.0	422	7(2%)	57.4 ± 19.5
chronic haematopoietic diseases	116	12(10%)	67.2 ± 16.2	189	20(11%)	71.8 ± 18.0
ophtalamic and laryngological	105	1(1%)	58.0 ± 16.9	160	0(0%)	61.8 ± 17.1
cancer	337	62(18%)	65.0 ± 12.8	277	56(20%)	66.2 ± 13.0
psychiatric	278	1(0%)	46.5 ± 18.2	143	0(0%)	52.3 ± 22.1
pulmonary	632	70(11%)	65.5 ± 17.2	534	50(9%)	70.6 ± 18.0
orthopedic	139	1(1%)	53.0 ± 17.4	187	0(0%)	59.6 ± 20.3
dermatological	47	2(4%)	54.6 ± 18.3	39	1(3%)	53.4 ± 20.1
n=	7336	580		6387	578	

## Statistical Analysis

In the statistical analysis, we used standard methods. For binary outcomes (i.e., acute deaths of ED patients), multivariable logistic regression was applied, with MCV and RDW exposures adjusted for age. The models were validated based on the Akaike information criterion (AIC). The statistical outcomes were expressed by classical odds ratios (ORs) together with 95% confidence intervals and p values. Additionally, we drew the probability curve of the mortality risk with age of the ED patients.

Receiver-operating characteristic (ROC) curves were used to choose the most appropriate threshold for a test of a binary classifier. The accuracy of the test was measured by the area under curve (AUC) [13]. The best thresholds for the highest true positive rate, together with the lowest false positive rate as well as the summary measure of the area under curve (AUC) in a ROC region of interest, are shown in the figures. In the statistical analysis, *p* values <0.05 were considered statistically significant.

The computation was performed on the R statistical platform [14]. The probability curve of acute mortality was displayed graphically using the “popbio” R package [15]. ROC analysis was performed using the pROC R package [16].

## Results

For the age of patients and the consecutive MCV and RDW categories, adjustment by age considerably improved the model fit, from AIC = 1619.114 for MCV and RDW alone to AIC = 1417.750 for the biomarkers and age together. The ORs of acute mortality (up to one month after the visit) in the ED (≥18) patients ages were first estimated using a multivariate logistic regression (Table 2).

**Table 2:** Odds ratios (ORs) MCV and RDW categories (adjusted by age) of acute mortality (until 1 month since the visit) in ED patients aged ≥18 with 95% confidence intervals and *p* values (multivariate logistic regression)

Risk factor	Level	Mortality	OR	CI95%	p value
Age	≥18	–	1.035	(1.031,1.038)	<0.0001
MCV [fL]	<80	8.6%	1.00	(ref.)	–
	75–79.9	6.7%	1.14	(0.94,1.40)	0.1839
	80–84.9	5.6%	1.39	(1.15,1.67)	0.0006
	85–89.9	6.5%	1.42	(1.17,1.72)	0.0003
	90–94.9	8.0%	2.17	(1.75,2.70)	<0.0001
	≥95	19.1%	2.60	(2.05,3.29)	<0.0001
RDW [%]	<13	3.0%	1	(ref.)	–
	13.0–13.4	4.8%	1.22	(1.03,1.43)	0.0174
	13.5–13.9	7.9%	1.61	(1.36,1.90)	<0.0001
	14.0–14.4	7.6%	1.46	(1.21,1.76)	0.0001
	14.5–14.9	11.4%	1.99	(1.67,2.38)	<0.0001
	15.0–15.4	13.2%	2.43	(1.97,3.00)	<0.0001
	≥15.5	19.2%	3.47	(2.99,4.03)	<0.0001

**Fig. 1:**
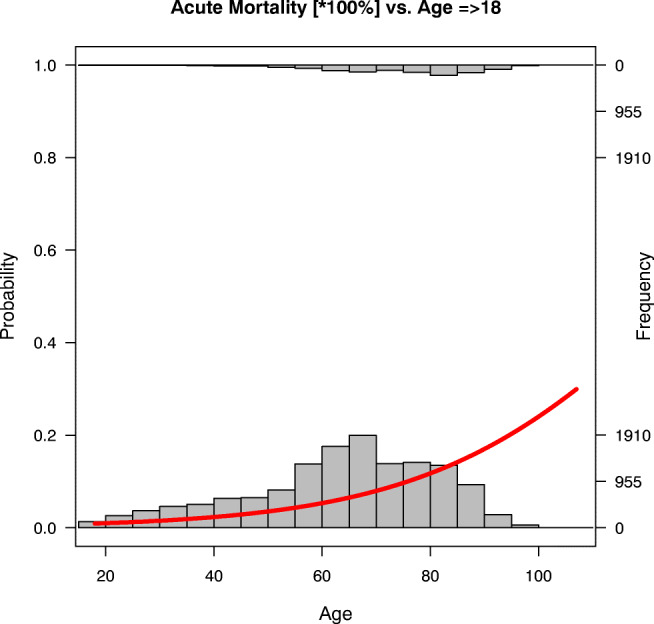
Model of acute mortality risk (until one month post-visit) in ED patients aged ≥18 (with frequency bars)

Statistical interpretation of the results reported in Table 1 is as follows: age is a statistically significant (p < 0.05) predictor of the acute death of patients; an increment of 10 years elevates the risk for early mortality up to (1.035^10^–1) × 100% ≈ 40%. The probability curve of the mortality risk (drawn in red) with (gray) frequency bars is shown in Fig. 1.

**Fig. 2:**
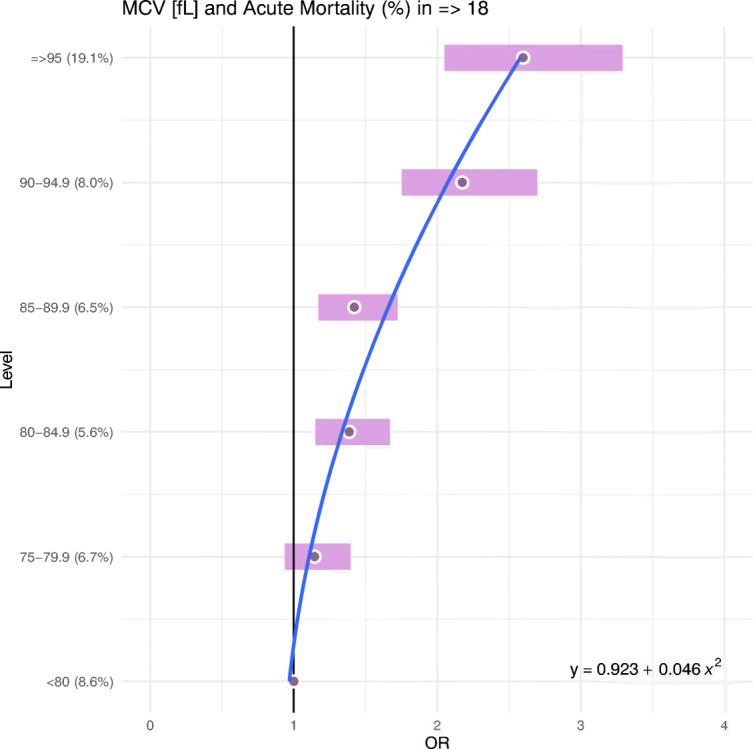
ORs for MCV categories (with quadratic approximation)

**Fig. 3:**
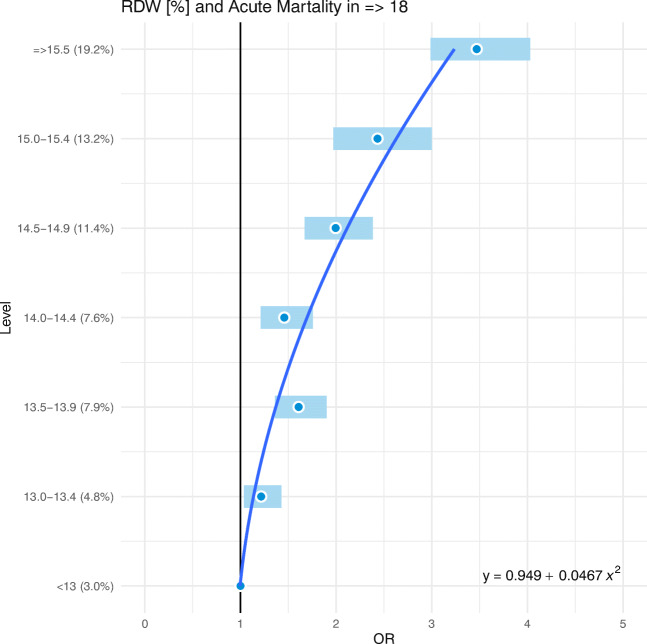
ORs for RDW categories (with quadratic approximation)

The ORs for the MCV and RDW categories are graphically displayed in Figs. 2 and 3.

Following Figs. 2 and 3, a typical U-shaped profile of the estimated ORs of the acute mortality in ED (≥18) patients can be established for MCV and RDW. Additionally, quadratic approximations of the ORs are reported in Table 3 and shown by (blue) solid curves (Figs. 2 and 3).

**Table 3:** Quadratic approximations of ORs (Table 1.)

Biomarker	Regression parameter	Mean	CI95%	*p*-value	R^2^
MCV [fL]	Intercept	0.92	(0.75,1.09)	0.0004	0.9637
	x^2^	0.05	(0.04,0.06)	0.0005	
RDW [%]	Intercept	0.95	(0.70,1.20)	0.0007	0.9473
	x^2^	0.05	(0.04,0.06)	0.0002	

From Table 2’s statistically significant (*p* < 0.05) regression parameter estimates and R-squared statistics, a strong fitting of the approximated quadratic models to the estimated ORs data can be ascertained, which confirms the U-shaped characteristic of the risk of acute mortality in ED (≥18) patients by their MCV and RDW. In addition, the estimated means’ regression parameters indicate a strong similarity between the statistical models (MCV ≈ RDW).

Finally, the prognostic values of the studied risk factors (MCV and RDW) were assessed using ROC analysis (Table 4).

**Table 4:** Prognostic values of age and MCV following ROC analysis

Risk factor	Threshold	Specificity [%]	Sensitivity [%]	AUC [%](CI 95%)
MCV [fL]	92.2	81.1	34.6	58.2(56.3–60.2)
RDW [%]	13.8	59.4	69.4	69.2(67.5–70.7)

**Fig. 4:**
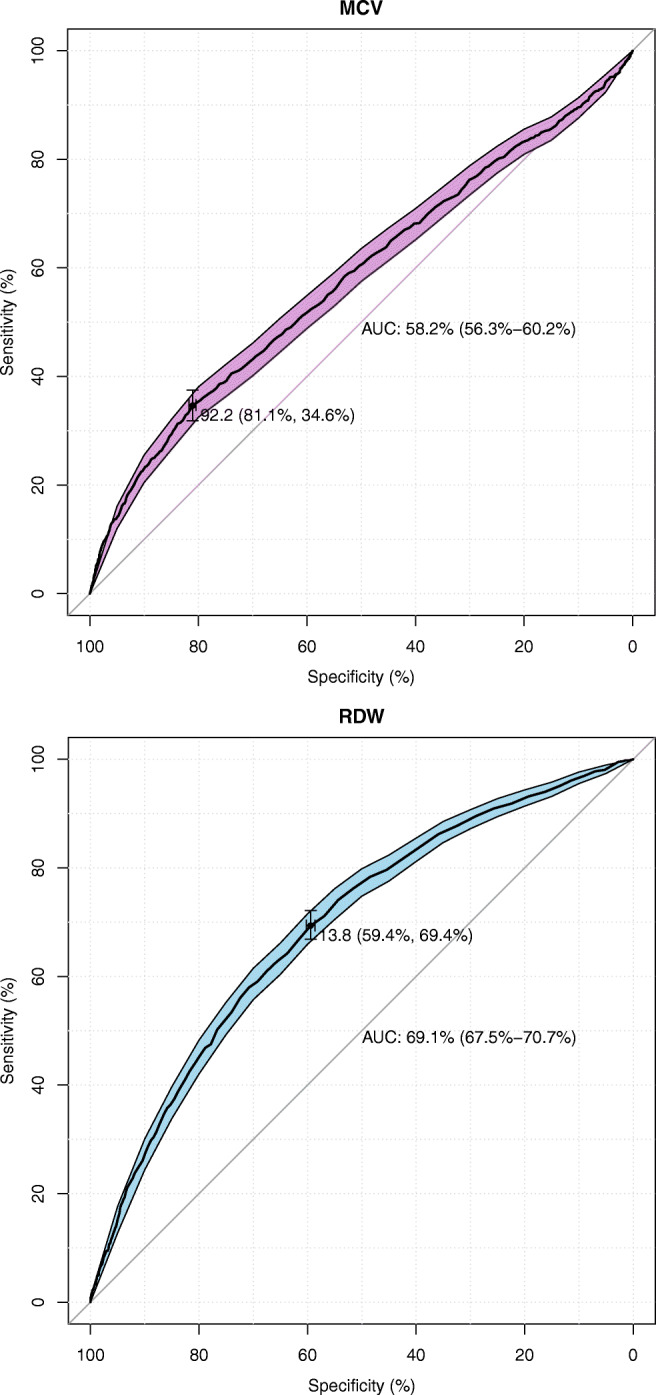
ROC curves with prognostic statistics of acute death of ED patients

After the ROC analysis reported in Table 4, it can be established that both MCV and RDW are statistically significant (AUC lower CI 95% >50%) predictors of acute mortality in ED patients. Classification of patients based on the MCV threshold = 92.2 units indicates the proper clinical prognosis in nearly 6 of 10 subjects (AUC >58%), whereas taking RDW (=13.8%) into account indicates a proper clinical prognosis in almost 7 of 10 individuals. The MCV characteristic suffers from its sensitivity rate being lower than its specificity: that is, using this scale, the test will be less able to correctly predict when death will occur, and more able to predict those who will still be alive. Much better sensitivity is calculated for the RDW biomarker (nearly 70%). This would mean that the majority of patients’ acute deaths would be correctly predicted. The ROC curves with the statistics described for MCV and RDW are presented graphically in Panels A and B, respectively, of Fig. 4.

**Fig. 5:**
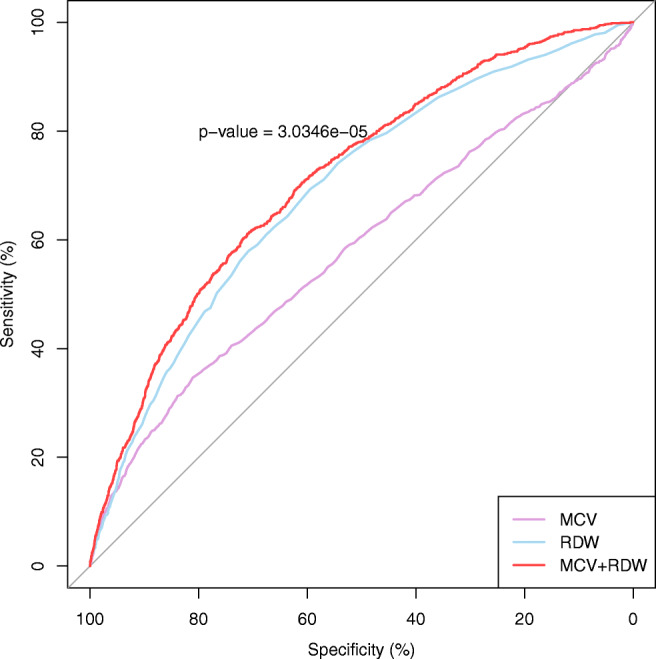
MCV, RDW, and MCV + RDW ROC curves (with a comparison test for RDW and MCV + RDW characteristics)

A combination of MCV and RDW predictors (MCV + RDW) did not strongly elevate the prognostic statistics of the clinical diagnosis of patients (AUC increased to 71.5%, nearly 2.5% compared to RDW alone), but the difference between the MCV + RDW (red) curve and the RDW (blue) characteristic (Fig. 5) remained statistically significant (*p* < 0.0001).

All the computations were performed in the R software [14]. The ROC curve analysis was performed using the “pROC” R package [16].

## Discussion

Aging is an inevitability of life and is most likely preprogrammed in the genes of all living organisms. It accelerates after reproductive age, when the genes have already been passed on to the next generation, and several mechanisms are currently proposed to accelerate this process. Based on animal studies and some human data, Bartke et al. propose that the elevated plasma level of growth hormone (GH) and IGF–I have a negative effect on life span [17]. In particular, low levels of circulating in blood IGF–I or somatomedin C, which is secreted from the liver in response to GH, has been shown to be a predictive marker of longevity in mice and humans. The clinical parameters affected by circulating IGF–I as a result of stimulation of erythropoiesis [12] are MCV and RDW [8, 9]. We were interested in whether these parameters could be used to predict acute mortality in older patients with chronic diseases treated in the ED. Herein we report that, by employing strongly fitting (95%) quadratic modeling of the ORs against studied biomarkers, we noticed a similar relationship between MCV and RDW as diagnostic tools to predict regenerative potential and clinical outcomes in older patients. Although RDW alone had a 10% higher diagnostic value in terms of predicting early death at EDs in patients who were admitted to the ED and subsequently hospitalized, the MCV measurement additionally improved the accuracy of predicting clinical outcomes by 2.5% compared to RDW alone. Based on this, we postulate that MCV and RDW could be potentially employed as relatively simple parameters to predict clinical outcomes with advanced age. Nevertheless, this intriguing conclusion requires further multicenter studies on a larger group of patients since we did not find in the literature any reports on multicollinearity between the MCV and RDW parameters.

It is well known that the response to tissue and organ damage as well as infections is mitigated by the coordinated pro-regenerative response of the stem cell compartment, which is confirmed by our observation. In fact, the number of circulating stem cells increases in the peripheral blood in several clinical situations, such as heart infarct, stroke, or systemic infection [18–21]. One stem cell population that, as postulated, plays an important role in tissue and organ regeneration and correlates in experimental animals with life span, is the population of very small embryonic-like stem cells (VSELs) [18, 22]. It has been reported that IGF–I has a negative effect on this pool of pluripotent/multipotent stem cells residing in adult tissues [11, 12]. To support the injection of IGF–I or upregulation of its expression decreases the life span of experimental animals 11,12, [22]. Therefore, it is likely that an increase in MCV and RDW parameters may correlate, in some of our older patients who have a poor prognosis, with an increased level of circulating IGF–I, which affects changes in red blood cell parameters [12]. This elevated level of IGF–I may over time lead to a decrease in the tissue pool of VSELs, and thus impair overall regenerative potential, leading to a worse prognosis during emergency situations. This intriguing hypothesis, however, requires further study.
